# Photochemistry of 2-thiooxazole: a plausible prebiotic precursor to RNA nucleotides†

**DOI:** 10.1039/d2cp03167a

**Published:** 2022-09-14

**Authors:** Lauren Bertram, Samuel J. Roberts, Matthew W. Powner, Rafał Szabla

**Affiliations:** a EaStCHEM School of Chemistry, University of Edinburgh, Edinburgh, UK; b Department of Chemistry, University College London, 20 Gordon Street, London, WC1H 0AJ, UK; c MRC Laboratory of Molecular Biology, Cambridge, UK; d Department of Physical and Quantum Chemistry, Faculty of Chemistry, Wrocław University of Science and Technology, 50-370 Wrocław, Poland

## Abstract

Potentially prebiotic chemical reactions leading to RNA nucleotides involve periods of UV irradiation, which are necessary to promote selectivity and destroy biologially irrelevant side products. Nevertheless, UV light has only been applied to promote specific stages of prebiotic reactions and its effect on complete prebiotic reaction sequences has not been extensively studied. Here, we report on an experimental and computational investigation of the photostability of 2-thiooxazole (2-TO), a potential precursor of pyrimidine and 8-oxopurine nucleotides on early Earth. Our UV-irradiation experiments resulted in rapid decomposition of 2-TO into unidentified small molecule photoproducts. We further clarify the underlying photochemistry by means of accurate *ab initio* calculations and surface hopping molecular dynamics simulations. Overall, the computational results show efficient rupture of the aromatic ring upon the photoexcitation of 2-TO *via* breaking of the C–O bond. Consequently, the initial stage of the divergent prebiotic synthesis of pyrimidine and 8-oxopurine nucleotides would require periodic shielding from UV light either with sun screening chromophores or through a planetary scenario that would protect 2-TO until it is transformed into a more stable intermediate compound, *e.g*. oxazolidinone thione.

## 1 Introduction

Proposing synthetic pathways to RNA and DNA nucleotides under prebiotically plausible conditions is of great importance for understanding the origins of life on Earth.^1–5^ The most selective reaction sequences involve periodic exposure to UV light, which enables the formation and purification of the biologically relevant ribonucleotides.^6–8^ Importantly, UV irradiation was suggested as a crucial environmental factor on Archean Earth due to the lack of atmospheric oxygen, in addition to a young Sun with slightly higher activity in the UV spectral range.^9^ Although atmospheric H_2_O and CO_2_ have demonstrated efficient shielding of wavelengths below 204 nm, high fluxes in the UV spectral region between 200 nm and 300 nm could have reached the prebiotic Earths surface due to the absence of UV-shielding molecular oxygen and ozone.^10,11^ Furthermore, UV light has proved to be an important source of energy, which can drive specific and selective chemical transformations, otherwise unattainable in the dark.^12,13^ For example, proposed syntheses leading to pyrimidine nucleosides and nucleotides as well as a complete set of Watson– Crick base-pairing arabino nucleotides, require UV light to enable key steps in the reactions sequences, such as photoanomerisation, photoreductions or desulfurisation.^7,8,14–19^

UV irradiation has also been suggested to be the primary selection factor on prebiotic Earth, as it leads to efficient photo-degradation of biologically irrelevant stereoisomers of nucleosides, in the reaction sequences proposed by Powner and co-workers.^6^ Moreover, isolated canonical nucleobases and nucleosides have been shown to undergo barrierless photo-relaxation mechanisms and exhibit very short excited-state lifetimes (at the level of ps) when compared to their non-canonical analogues, which is directly related to their generally higher photostability.^20–27^ Therefore, photostability of A, G, C, T and U was previously thought to be one of the key reasons why biology selected these particular building blocks as fundamental RNA and DNA components.^24^ However, in an aqueous prebiotic environment, pyrimidine nucleosides may undergo photohydration reactions, which in addition to photodimerisation of pyrimidine bases in oligomers poses some challenges to prebiotic photochemistry.^6,28,29^

Most recent results suggest that pyrimidine nucleosides can be partly protected from complete photohydration by varying temperature and day-night cycles or potentially sun-screening with more photostable chromophores.^30,31^ In addition, DNA oligomers can undergo UV-induced self-repair in more photostable sequences or with the support of electron-donating nucleobase analogues, such as 2,6-diaminopurine or 8-oxo-guanine.^32,33^ Nevertheless, it is still unclear whether many of the prebiotic precursor molecules to nucleosides were photostable enough to enable efficient formation of RNA building blocks in UV-rich prebiotic environments.

**Fig. 1:**
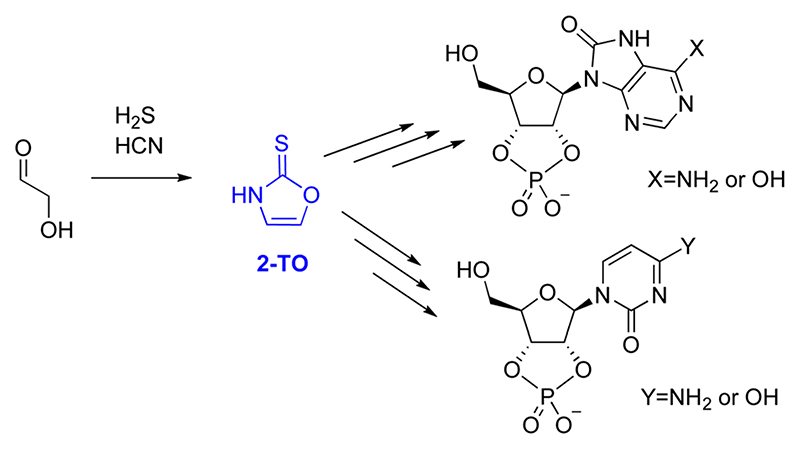
General scheme to 8-oxo-purine ribonucleotides (top) and a pyrimidine ribonucleotides (bottom) from glycolaldehyde, through the small organic molecule intermediate 2-thiooxazole, 2-TO (shown in blue).

The majority of proposed selective syntheses to RNA monomers either account for the formation of pyrimidine^6,34^ or purine^3^ ribonucleotides but fail to describe the formation of both simultaneously. Therefore, in 2017, Stairs *et al*. suggested a divergent synthetic route, leading to pyrimidine and 8-oxo-purine nucleotides.^35^ These reaction sequences proceed from glycolaldehyde through one of two small organic molecules, either 2-aminooxazole (2-AO) or 2-thiooxazole (2-TO), with an example for 2-TO shown in Fig. 1.

2-AO has previously been suggested as a possible intermediate in several plausible pathways to pyrimidine ribonucleotides^6,36,37^ and in the synthesis of all four Watson–Crick arabino nucleotides.^15^ Therefore, its photochemistry and photostability has been studied extensively, by both theoretical chemistry^38,39^ and UV irradiation experiments,^40^ to assess its viability as a plausible precursor. These results demonstrated that even though 2-AO undergoes fast photodegradation in an aqueous environment, it can be partially protected from UV light by the presence of more photostable chromophores created under the same conditions, such as 2-aminoimidazole (2-AI), through sun screening.^40,41^ However, the photochemical properties of 2-TO have not been investigated yet and it remains unclear whether it is sufficiently photostable to accumulate under UV-irradiation conditions.

In this work, we have conducted computational studies and UV irradiation experiments to gain insight into the photochemistry of 2-TO when irradiated with UV light. In particular, our experimental results suggest that 2-TO undergoes efficient photodegradation leading to similar photoproducts as in the case of other related oxazole molecules. We explain this behaviour based on nonadiabatic excited-state dynamics simulations, which show that 2-TO may efficiently populate triplet excited states in a similar way as thionucleobases^42–53^ and indicate the underlying photodegradation mechanisms. These results impose further constraints on prebiotic scenarios leading to the formation of RNA nucleotides in UV-rich environments.

## 2 Methodology

### 2.1 Experimental methods

A solution of 2-thiooxazole, 2-TO, (6.00 mmol, 2 mM in H_2_O) was adjusted to the desired pH (pH 3, 6.5 or 12 for irradiation at 254 nm and pH 6.5 for irradiation at 300 nm) and then degassed with a stream of nitrogen for 2 h in a quartz tube. The tube was sealed and kept under a positive argon atmosphere. The solution was irradiated at 254 or 300 nm for 16 h at 38 °C, then left to relax for 1 h. The solution was analysed by NMR spectroscopy. A solution of pentaerythritol (0.100 M, 50.0 μL, 5.00 μmol in H_2_O/D_2_O 9 : 1) was added as an internal NMR standard and the NMR spectra were then reacquired. Analysis of the NMR spectra showed consumption of starting material in all cases (except 300 nm, pH 6.5, 29% 2-thiooxazole) and no formation of oxazole. NMR and UV-vis spectra of thiooxazole are presented in Fig. S6–S8 in the ESI.†

### 2.2 Computational methods

The ground-state equilibrium geometry of 2-TO was optimised at the MP2/aug-cc-pVTZ level of theory.^54,55^ This geometry was used for calculations of the vertical excitation energies. Vertical excitation energy calculations and geometry optimisations of the excited state minima (S_2_, S_1_, T_2_ and T_1_) were all performed using the algebraic diagrammatic construction method to the second-order method [ADC(2)]^56–58^ with the aug-cc-pVTZ correlation consistent basis set. Optimisations of all relevant minimum-energy crossing points (MECPs) between different electronic states were performed using the method implemented by Levine *et al*., which allows the optimisation of MECPs without the evaluation of nonadiabatic couplings.^59^ The energies and analytical gradients were computed at the MP2 and ADC(2) levels for the electronic ground and excited states respectively, employing the aug-cc-pVTZ basis set. Our in-house implementation of this optimisation scheme^60^ was coupled with the Turbomole 7.5 package.^61^

Simulations of the UV-vis spectrum and the semi-classical nonadiabatic molecular dynamics were performed at the ADC(2) level with the aug-cc-pVDZ basis set (in the gas phase). For this purpose, we also used the ground-state geometry and vibrational normal modes obtained at the MP2/aug-cc-pVDZ level. Our results demonstrate that this basis set offers sufficiently accurate structures and excited-state energies and allows to reduce the high computational cost of nonadiabatic molecular dynamics simulations.

The nonadiabatic molecular dynamics simulations were performed for isolated 2-TO using Tully’s fewest switches surface hopping algorithm^62^ with the energy-based decoherence scheme of Granucci and Persico, which uses a decoherence parameter of 0.1 Hartree.^63^ The UV-vis absorption spectrum was simulated using the excitation energies and oscillator strengths from single point calculations of 500 initial geometries. These initial geometries were obtained by sampling from a Wigner distribution for all vibrational normal modes of the ground-state geometry. The four lowest lying singlet states and three lowest lying triplet states were considered for the absorption spectrum as well as the trajectories in the non-adiabatic molecular simulations. Initial conditions for the nonadiabatic molecular dynamics simulations were selected from the excitation window of 4.5–4.7 eV, *i.e*. around the simulated absorption maximum of 2-TO. This excitation window also overlaps with the third harmonic generation of the Nd:YAG laser (266 nm), which is broadly used as an excitation source in time-resolved pump probe experiments and the results of our simulations can be compared to such experiments in the future. This excitation window resulted in 141 initial conditions for the trajectories, with 5 starting in the S_1_ state, 120 in the S_2_ state and 16 in the S_3_ state. The trajectories were propagated for up to 1000 fs with a 0.5 fs time step for the nuclei and a time step of 0.02 fs for the electronic time-dependent Schrödinger equation.

The trajectories were analysed up to the point where the energy gap between the ground-state and the first populated excited state, either S_1_ or T_1_, dropped below 0.15 eV, as the ADC(2) method becomes unreliable near the area of conical intersections with the ground-state.^64^ When this criterion is met, it is assumed that if the current populated state is S_1_ then a surface hop to S_0_ will occur and the molecule will remain in the ground-state until the end of the trajectory. However, if the current populated state is T_1_ when the criterion is fulfilled, it is assumed that the molecule remains in T_1_ until the end of the trajectory. For this analysis, 2 trajectories were ignored as they faced problems with ADC(2) convergence. This approach allows us to identify the possible photorelaxation channels of 2-TO, even though the lack of nonadiabatic (or spin-orbit) couplings and surface hoppings between either the S_1_ or T_1_ states and the S_0_ state does impose some limitations.

The potential energy (PE) profiles were constructed by linear interpolation in internal coordinates (LIIC) between the S_0_ minimum energy geometry, minimum energy geometries of excited states involved in a given photorelaxation pathway (S_1_, T_2_ or T_1_) and the corresponding state crossings between them for all photorelaxation pathways apart from when NH bond fission occurs. This pathway was constructed by LIIC between the S_0_ minimum energy geometry and the state crossing associated with NH bond fission, following the πσ^*^ state. For all PE profiles, the energy was plotted against mass-weighted coordinates. The energies were calculated with the ADC(2) and MP2 methods and the aug-cc-pVTZ basis set for all intermediate geometries and all photorelaxation channels, apart from the channel that lead to the C(2)–O bond breaking in the singlet state. The potential energy profile for this latter channel was mainly constructed by LIIC between the S_0_ and S_1_ minima, and we subsequently performed a relaxed scan for the C(2)–O bond breaking process from the S_1_ minimum to reach S_1_/S_0_ state crossing. Owing to the instability of the ADC(2) method along this relaxed scan, the energies of the interpolated geometries as well as the relaxed scan for the C(2)–O bond breaking mechanism were obtained using the SCS-ADC(2)/aug-cc-pVTZ method.^65^ Nevertheless, our subsequent calculations performed at the (regular) ADC(2) level revealed that it can reproduce a smooth potential energy surface for this process, which is necessary for nonadiabatic molecular dynamics simulations. The analogous relaxed scan performed for the C(2)–O bond breaking mechanism from the T_1_ minimum-energy geometry returned a smooth PE profile with the regular ADC(2) approach and, thus, SCS-ADC(2) was not applied in this case.

The accuracy of the ADC(2) methods was validated with energy calculations performed for all PE profiles mentioned above (using the same geometries) at the NEVPT2/SA-CASSCF(10,8) level^66^ with a cc-pVTZ basis set,^67^ apart from the NH bond fission pathway, where an augmented basis set, aug-cc-pVDZ, is required to describe the Rydberg-like repulsive πσ* state.^68^ In these calculations, the SA-CASSCF wave-function was averaged over the 6 lowest-lying singlet states and the 6 lowest-lying triplet states. The active space was composed of 10 electrons correlated in 8 orbitals (4π, 1n_S_, 2π^*^ and $11\sigma_{\text{NH}}^{*}$). The choice of this active space was based on the rules proposed by Veryazov *et al*., who suggested that the molecular orbital occupations of active orbitals should be kept in the range of 0.02–1.98.^69^

All the MP2 and ADC(2) calculations (including the SCS variants) were performed using the Turbomole 7.5 package.^61^ Computations of the UV-vis absorption spectrum and the nonadiabatic molecular dynamics were performed with the SHARC 2.1 method^70–73^ interfaced with Turbomole 7.5 and Orca 4.2.1.^74^ The NEVPT2/SA-CASSCF calculations were performed using the Orca 4.2.1 package.^74^

## 3 Results and discussion

### 3.1 UV-irradiation experiments

To evaluate the photostability of 2-TO, samples of the compound were dissolved in water at neutral pH and exposed to continuous irradiation either at 254 nm or at 300 nm, for 16 h (ESI,† Fig. S4). Exposure to UV light at both wavelengths resulted in complete consumption of the starting material leading to a complex mixture of small molecule unknown photoproducts. It is worth noting that although the NMR spectra of the product mixture indicate the formation of small molecule photoproducts (see Fig. S4 and S5 in the ESI†), we were not able to clearly assign the signals to particular compounds. Alkaline pH had negligible effects on the results and formed photoproducts. Our previous work showed that 8-mercaptopurine nucleosides (containing a thiocarbonyl group as a part of the imidazole fragment of the purine base) desulfurise under similar irradiation conditions.^15^ A similar process in 2-TO would lead to the formation of oxazole, which was not detected among the photoproducts formed during UV irradiation of 2-TO. This observation is consistent with 2 mechanisms, either that oxazole undergoes rapid photodegradation after it is formed or that 2-TO itself follows an entirely different photodegradation mechanism, which does not involve the formation of the oxazole intermediate through desulfurisation. To further investigate this ambiguity, oxazole was exposed to 16 h of UV irradiation at 254 nm, which also resulted in complete degradation of the starting material. The NMR spectra acquired for the mixtures of photoproducts in fact indicate that some common photoproducts might be formed during the irradiation of both 2-TO and oxazole (ESI,† Fig. S5). It is worth noting that, oxazole and its derivatives were previously shown to undergo C–O bond breaking upon the exposure to UV light.^39,75^ Nevertheless, these experimental results did not offer a clear answer whether 2-TO can undergo such a direct photochemical disruption of the aromatic ring or its photochemistry is initiated with sulphur loss. Therefore, we further performed accurate quantum chemical and nonadiabatic excited-state dynamics simulations to determine the mechanism of 2-TO photodegradation.

### 3.2 Ground state geometry and excited state minima

**Fig. 2:**
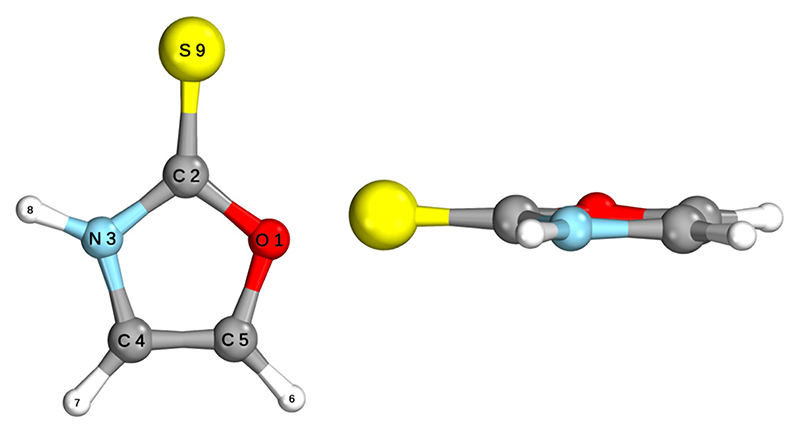
Ground state geometry of 2-TO, from two angles, optimised at the MP2/aug-cc-pVTZ level.

**Fig. 3:**
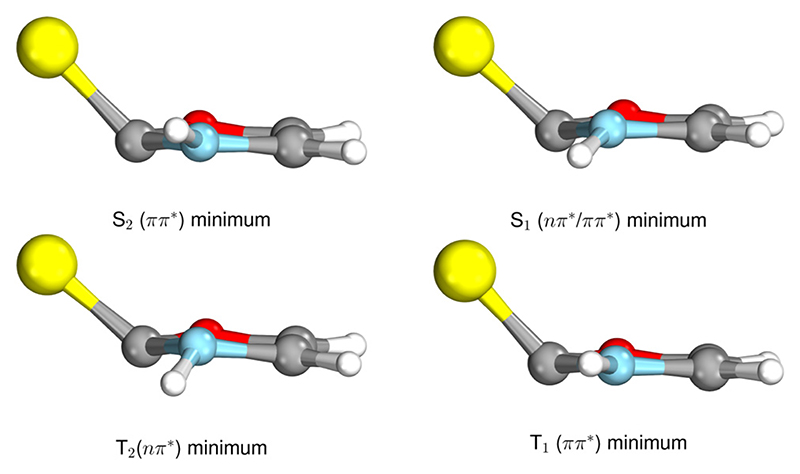
Geometries of the excited state minima, S_2_, S_1_, T_2_ and T_1_, computed at the ADC(2)/aug-cc-pVTZ level.

The ground state geometry of 2-TO, optimised at the MP2/aug-cc-pVTZ level in the gas phase, is shown in Fig. 2. 2-TO has a planar ring structure and the thiocarbonyl C=S bond (1.64 Å in length) is also planar with respect to the ring in the electronic ground state. The excited state minima (S_2_, S_1_, T_2_ and T_1_) were optimised at the ADC(2)/aug-cc-pVTZ level and are shown in Fig. 3. Dissimilar to the ground state equilibrium geometry, all four of these excited state minima are characterised by elongation of the C–S bond (by approximately 0.13 Å) and its displacement (tilting) out of the aromatic ring plane. Elongation and displacement of this bond occurs due to these states having either ${\text{ππ}}_{\text{CS}}^{*}$ or ${\text{nπ}}_{\text{CS}}^{*}$ molecular orbital character, where these orbitals are mainly located on the thiocarbonyl group. These structural changes are the result of the promotion of an electron to the ${\text{π}}_{\text{CS}}^{*}$ molecular orbital, which is antibonding with respect to the C=S bond (see Fig. S2 in the ESI† for the associated molecular orbitals). All of the optimised excited state minimum-energy geometries are also characterised by slight puckering of the aromatic ring which is most pronounced at the thiocarbonyl C(2) atom for the S_2_ and T_1_ minimum-energy structures (${\text{ππ}}_{\text{CS}}^{*}$ character) and the N heteroatom for the T_2_ (${\text{nπ}}_{\text{CS}}^{*}$ character) and S_1_ (${\text{nπ}}_{\text{CS}}^{*}/{\text{nπ}}_{\text{CS}}^{*}$mixed character) structures.

### 3.3 Vertical excitation energies and UV-vis spectrum

Vertical excitation energies were computed for 2-TO in the gas phase as well as using the conductor-like screening model (COSMO) as an implicit solvation model of bulk water (see Table 1). In the gas phase, the S_1_ state has an excitation energy of 4.23 eV and is a dark ${\text{nπ}}_{\text{CS}}^{*}$ state, which is associated with an excitation from the lone electron pair on the S atom into the π^*^ orbital primarily located on the thiocarbonyl group. This state is characterised by very low oscillator strength and we anticipate that its direct population through UV absorption is negligible. The optically bright S_2_ state lies ~0.4 eV above the dark S_1_ $\left(n{\text{π}}_{\text{CS}}^{*}\right)$ state and has the ${\text{ππ}}_{\text{CS}}^{*}$ molecular orbital character, with the ${\text{π}}_{\text{CS}}^{*}$ situated predominantly on the thiocarbonyl group (see Fig. S1 in the ESI† for the associated molecular orbitals). The πσ^*^ and nσ^*^ states, located above S_2_, indicate that there may be potentially accessible photorelaxation pathways involving N–H bond fission during the excited state dynamics simulations.

In the presence of the conductor-like screening model (COSMO) of bulk water, we observe a reordering of the first two excited states, when compared to the gas phase computations.

The ${\text{nπ}}_{\text{CS}}^{*}$ state is significantly blue-shifted (by ~ 0.4 eV) relative to the gas phase value, and the ${\text{ππ}}_{\text{CS}}^{*}$ state is slightly red-shifted.^76^ Consequently, the order of these states is reversed and the S_1_ state now becomes the bright ππ^*^ state. While the solvent model also has a pronounced effect on the vertical excitation energies of the S_3_–S_6_ states, resulting in hypsochromic shifts between 0.3 and 0.5 eV, their ordering remains unchanged. Therefore, the optically bright S_2_ state is well separated from other excitations with sizeable oscillator strengths in an aqueous environment and will be the primary electronic transition of 2-TO responsible for the absorption of light in the UV-B spectral region. The bathochromic shift of the S_2_ $\left({\text{ππ}}_{\text{CS}}^{*}\right)$ state is the direct result of the electric dipole moment vector *μ* having very similar direction and somewhat larger magnitude (6.32 D) when compared the the *μ* vector of the S_0_ electronic state (5.71 D; see Tables S_1_ and S_2_ in the ESI†). In contrast, the directions of the *μ* vectors of the remaining excited singlet states presented Table 1 are very different from the *μ* vector of the ground state, which results in their hypso-chromic shift.

**Fig. 4:**
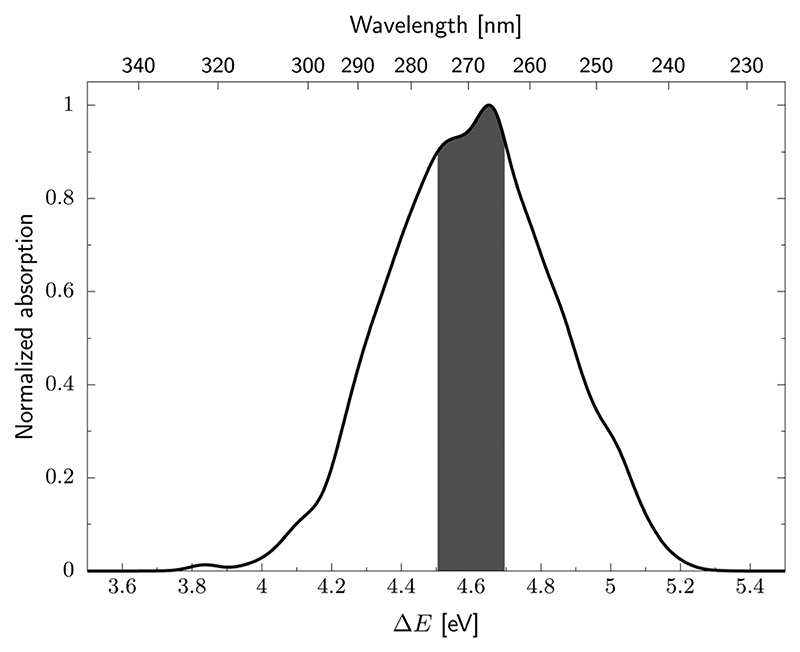
UV absorption spectrum of 2-TO simulated at the ADC(2)/aug-cc-pVDZ level. The excitation window for the dynamics is shown by the shaded area of the spectrum.

We next simulated the UV-vis absorption spectrum in the gas phase using the ensemble approach, which is presented in Fig. 4.^77^ This spectrum is characterised by one strong absorption band with a maximum around 4.65 eV, which is consistent with the dominant absorption originating from the S_2_ $\left({\text{ππ}}_{\text{CS}}^{*}\right)$ electronic state. There are overlapping contributions from the neighbouring S_1_ and S_3_ states, which contribute to the broadening of this band. To follow the excited state dynamics of 2-TO, an excitation window of 4.5–4.7 eV was chosen (shown by the shaded area in Fig. 4), as this corresponds to excitation around the peak maximum. This spectral window should also correlate well with our UV-irradiation experiments, since it ensures dominant excitation to the ${\text{ππ}}_{\text{CS}}^{*}$ electronic state (as expected for aqueous environment) and allows for the generation of sufficiently large ensemble of initial conditions for the subsequent nonadiabatic molecular dynamics simulations.

### 3.4 Nonadiabatic molecular dynamics simulations

We next performed surface hopping excited-state simulations of isolated 2-TO including the four lowest-lying singlet and three lowest-lying triplet states using the ADC(2)/aug-cc-pVDZ method for electronic structure calculations. Given the very high intersystem crossing yields in other aromatic chromophores containing thiocarbonyl groups, our simulations also accounted for radiationless transitions (hoppings) between the singlet and triplet manifolds of electronic states. As expected, selection of initial conditions from the spectral window of 4.5–4.7 eV resulted in 85% of the trajectories being initiated in the S_2_ state with 11% and 4% of the trajectories starting in the S_3_ and S_1_ states, respectively. It is worth noting that for different displaced geometries generated from the Wigner distribution, the ordering of electronically excited states might be interchanged and it will not always reflect the vertical excitation energies calculated for the equilibrium geometry (Table 1). Thus, the vast majority of our trajectories (~89%) were initiated in the ${\text{ππ}}_{\text{CS}}^{*}$ state.

**Fig. 5:**
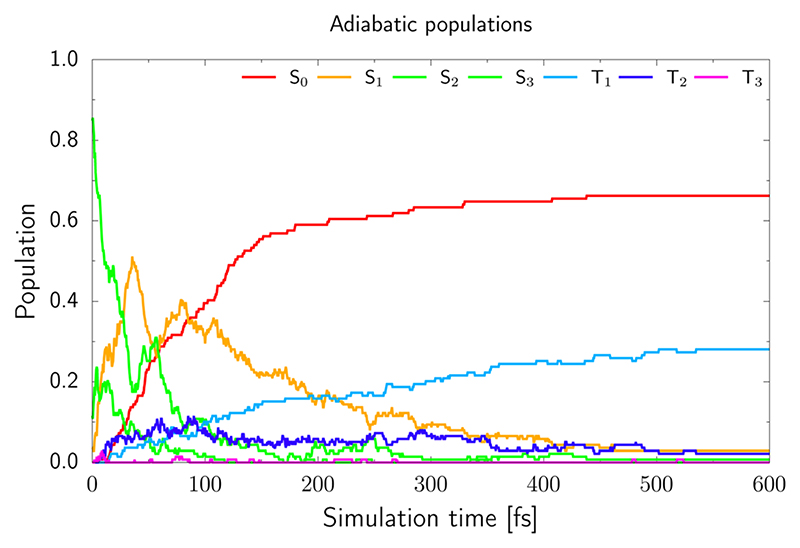
The adiabatic populations of 2-TO of the ground and excited states over the time of the trajectories.

From the adiabatic populations shown in Fig. 5 we see that the excited state population is very quickly transferred into the S_1_ state. Subsequently, the trajectories either undergo direct photorelaxation to the S_0_ state, or intersystem crossing to the triplet manifold, which occurs predominantly by the S_1_ → T_2_ pathway. This is reflected by the increasing and larger population of the T_2_ state during the first 90 fs of the simulation, which also exceeds the population of the T_1_ state during the period. After 90 fs, the T_2_ population decreases and the T_1_ population increases, indicating efficient relaxation to the T_1_ state from the higher-lying triplet state. The evaluation of hoppings to the S_0_ state would be relatively inaccurate with the ADC(2) method, as the method becomes unreliable in areas near conical intersections with the ground state. Therefore we have assumed that once the energy gap between the S_1_ and S_0_ states drops below 0.15 eV, for a given trajectory, the S_0_ state is repopulated. However, the hopping probability between the T_1_ and S_0_ states is strongly dependent on spin–orbit couplings and we cannot include the repopulation of the S_0_ state from the T_1_ state in our model. Therefore, all trajectories which reached the T_1_ electronic state were terminated when the energy gap between the T_1_ and S_0_ states dropped below 0.15 eV, but we assumed that the excited molecule stayed in the triplet manifold. Consequently, the population of the S_0_ state presented in Fig. 5 only reflects the cumulative contribution of the singlet S_1_ → S_0_ photorelaxation channel and only in an approximate manner. This also does not allow us to draw clear conclusions about the estimated excited-state lifetime of 2-TO. Taking into account the above assumptions and limitations of our approach, our simulations resulted in 68% of the trajectories undergoing direct photorelaxation in the singlet manifold. The remaining 32% of the trajectories accessed the triplet manifold *via* intersystem crossing.

After more detailed analysis we classified the simulated trajectories into six photorelaxation channels, four in the singlet manifold and two in the triplet manifold. The dominant pathway is a photodestructive deactivation channel and has been located in both the singlet and triplet states. This channel is associated with the C(2)–O bond breaking and is activated after population of the repulsive $\text{π}\sigma_{\text{CO}}^{*}$ state, which is accessible in both the S_1_ and T_1_ states. Approximately, 50% of trajectories proceeded through this channel, with 80% of these in the singlet manifold and 20% in the triplet manifold. The dominant photostabilising channel, which was followed by 37% of the trajectories, is associated with C–S bond tilting and aromatic ring puckering due to the pyramidalisation of the N atom. This mechanism may lead to an n_S_π^*^/S_0_ MECP in both the singlet and triplet manifolds. The remaining 13% of trajectories were deactivated through the N–H bond fission pathway, in the singlet state, which is driven by the repulsive $\text{π}\sigma_{\text{NH}}^{*}$ state.^68^ There was one additional photodestructive channel followed by a single trajectory, leading to πσ^*^/S_0_ MECP associated with the breaking of the C(5)–O bond. As only one trajectory followed this pathway, we anticipate that this channel may have an insignificant impact on the overall photodeactivation of 2-TO (PE profiles for these minor photorelaxation mechanisms are presented in Fig. S3 in the ESI†).

**Fig. 6:**
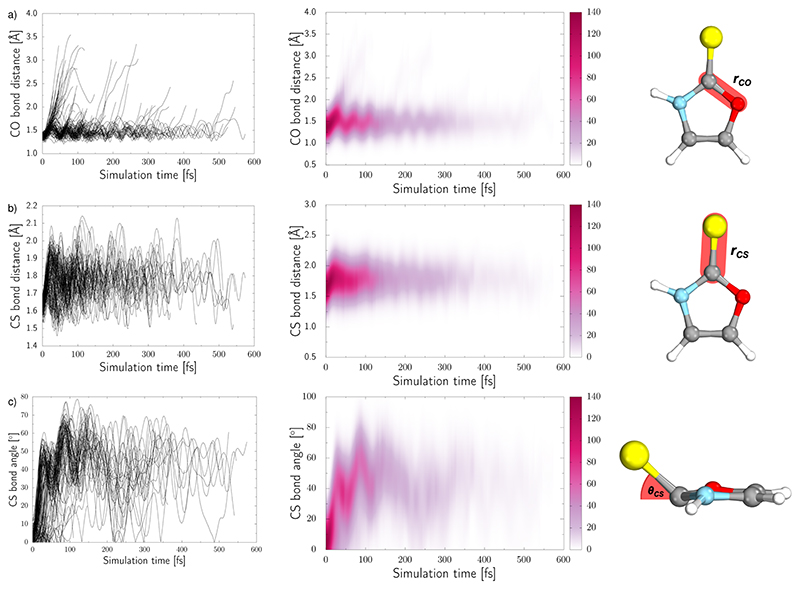
The time-dependence of three internal coordinates of 2-TO (shown on the right) throughout the nonadiabatic dynamics simulations: (a) C–O bond distance; (b) C–S bond distance; (c) angle between the C–S bond and the plane of the aromatic ring. Each trajectory is represented by a single line in the plots on the left.

Three internal coordinates characteristic of the dominant photorelaxation pathways were monitored over the time of these excited state dynamics simulations for each of the trajectories and are shown as both line and convolution plots in Fig. 6. The first internal coordinate (a), the C(2)–O bond distance, demonstrates the number of trajectories that followed this photodestructive channel and times at which the C(2)–O bond dissociation occurred. Initially, the majority of trajectories oscillate around the equilibrium C(2)–O bond distance in the ground state of 1.37 Å. However, it is also evident that a group of over 20 trajectories accessed the dissociative $\text{π}\sigma_{\text{CO}}^{*}$ state in a ballistic manner during the first 50 fs of the simulation. This is the result of the excess energy acquired by 2-TO during the photoexcitation and occurred for the initial conditions with the C(2)–O bond stronger displacement out of its equilibrium distance. However, majority of the trajectories which resulted in C(2)–O bond breaking underwent at least several oscillations around the equilibrium bond length before approaching the the $\text{π}\sigma_{\text{CO}}^{*}/{\text{S}}_{0}$ state crossing. Trajectories that start to exceed a bond distance of 1.8 Å terminated shortly after, indicating the molecule has accessed the πσ^*^/S_0_ MECP, associated with the C(2)–O bond breaking.

In panel (b) of Fig. 6, the C–S bond distance has been plotted over time, as all relevant excited state minima and MECPs between excited states correspond to an elongation of this bond. During the dynamics the average bond distance very quickly increased from the equilibrium ground-state bond distance of 1.64 Å and the associated C–S oscillations are characterised with very high amplitudes (1.5–2.1 Å). As described above for the excited-state minima, this elongation is the consequence of the biradical character of the ${\text{ππ}}_{\text{CS}}^{*}$ and ${\text{nπ}}_{\text{CS}}^{*}$ electronic states both in the singlet and triplet manifolds.

In addition to the C–S bond lengthening in the excited state minima and MECPs between excited states of nπ^*^ and ππ^*^ character, the angle between the plane of the aromatic ring and thiocarbonyl group also increases from the planar geometry in the ground-state. This angle variation throughout the trajectories can be seen from the plots presented in panel (c) of Fig. 6. Similarly to the C–S bond elongations, the C–S tilting motion occurs in a barrierless manner reaching high magnitude of this angle (over 40°) already after initial 50 fs. The C–S bond angle also exhibits oscillations with very high amplitudes, that is between 10° and 80°, which continue until the photorelaxation of each trajectory occurs. It is worth noting that we have presented the absolute value of this angle for simplicity.

### 3.5 Potential energy profiles for the dominant photorelaxation channels

**Fig. 7:**
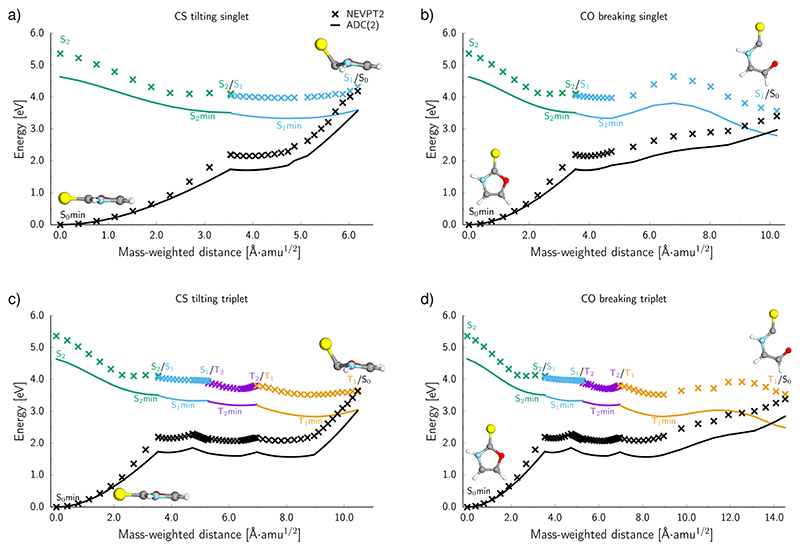
The potential energy (PE) profiles of the four main deactivation channels of 2-TO calculated at the MP2/ADC(2) level for (a, c and d) and at the SCS-MP2/SCS-ADC(2) level for (b), all with an aug-cc-pVTZ basis set (solid lines). Crosses correspond to the energies calculated at the NEVPT_2_/SA-CASSCF/cc-pVTZ level. The minimum energy crossing points with the ground state were located from the nonadiabatic dynamics simulations. The PE profiles have been interpolated between excited state minima and minimum energy crossing points that were involved in these pathways.

To further characterise the radiationless deactivation mechanisms from the nonadiabatic dynamics simulations, we constructed potential energy (PE) profiles by linear interpolation in internal coordinates between the S_0_ equilibrium geometry, the relevant excited state minima and the corresponding minimum energy crossing points (MECPs). The PE profiles were plotted for each of the six photorelaxation channels identified in the dynamics simulations and the four most frequent channels, two photodestructive and two photostabilising, are shown in Fig. 7. These energies were computed at the ADC(2)/MP2 level for pathways (a, c and d) and at the SCS-ADC(2)/SCS-MP2 level for pathway (b) (see the Methods section for more details), and were plotted against mass weighted coordinates for each of the photorelaxation pathways.

The pathways are shown from the S_2_ state in the Franck–Condon region, as 85% of trajectories initially started on this state and it is assumed that trajectories starting in S_3_ would follow a barrierless transition onto S_2_. Following excitation into the bright ${\text{ππ}}_{\text{CS}}^{*}$ state, the molecule relaxes into the very shallow S_2_ minimum, with the same molecular orbital character as in the Franck–Condon region. Once in the S_2_ minimum, the molecule has almost a barrierless transition to access the S_2_/S_1_ MECP that allows for internal conversion (IC) into the S_1_ state and the associated ${\text{S}}_{1}\left({\text{nπ}}_{\text{CS}}^{*}\right)$ minimum. Then from this S_1_ minimum, the molecule either continues in the singlet manifold and accesses a S_1_/S_0_ MECP or it crosses into the triplet manifold through intersystem crossing (ISC). It is worth noting here, owing to the excess energy acquired by 2-TO during a photoexcitation event most trajectories never reach the near vicinity of the S_1_ minimum, but oscillate around it to undergo subsequent IC to the ground state or ISC to the triplet manifold. However, since the presented PE cross sections pass through the lowest energy points on specific excited-state surfaces, they demonstrate the extreme case and minimum amounts of energy necessary to follow a given photodeactivation mechanism.

In the photostabilising singlet channel in Fig. 7(a), where the C–S bond tilts out of the plane of the aromatic ring and the ring puckers due to pyramidalisation of the N atom, the molecule has a small barrier of 0.3 eV to overcome from the S_1_ minimum, in order to access the ${\text{nπ}}_{\text{CS}}^{*}/{\text{S}}_{0}$ MECP, which is predicted to be a relatively facile process as a number of trajectories followed this pathway. The photodestructive channel in the singlet state (b), has a slightly higher energy barrier of 0.4 eV between the S_1_ minimum and the $\text{π}\sigma_{\text{CO}}^{*}/{\text{S}}_{0}$ MECP associated with the C(2)–O bond breaking. However, as demonstrated through the excited-state dynamics simulations, 2-TO excited above 4.5 eV has sufficient amounts of excess vibrational energy to readily overcome this barrier. This is manifested by the singlet C(2)–O bond breaking pathway being the dominant photorelaxation channel.

The two reaction pathways that occur in the singlet state also occur in the triplet manifold and are shown in Fig. 7 (where (c) is the photostabilising pathway and (d) is the photodestructive pathway). These channels are both accessed *via* a S_1_/T_2_ MECP followed by relaxation into a shallow T_2_ (n_S_π_*_) minimum. Subsequent relaxation into the T_1_ (ππ^*^) minimum occurs readily through the T_2_/T_1_ MECP. From the T_1_ minimum, the molecule must overcome a modest barrier of 0.2 eV to access the ππ^*^/S_0_ MECP, associated with the photostabilising pathway (c), where the C–S bond tilts out of the plane. The photodestructive channel (d) also has a barrier of 0.2 eV from the T_1_ minimum to the πσ^*^/S_0_ MECP that involves the C(2)–O bond breaking. Consequently, the triplet manifold has easily accessible MECPs for both the photostabilising and photodestructive channels.

### 3.6 Benchmarking ADC(2) potential energy surfaces against the NEVPT2 method.

To evaluate the accuracy and reliability of the dynamics and PE profiles computed at the ADC(2) level, we performed additional single point calculations for the primary photorelaxation channels using the NEVPT_2_/SA-CASSCF(10,8) approach with the cc-pVTZ basis set (see Fig. 7). These calculations were carried out for each of the interpolated geometries in an analogous way as for PE profiles calculated at the ADC(2) level of theory. Qualitatively, the shapes of the PE surface cuts for the four main deactivation channels, shown in Fig. 7, are consistent between the ADC(2) and NEVPT(2) methods. In general, the excited state energies as well as the energies of the S_1_/S_0_ and T_1_/S_0_ MECPs are systematically higher by approximately 0.6 eV at the NEVPT2 level, when compared to the ADC(2) results. However, the position of the MECPs with the S_0_ state relative to the mass-weighted coordinates is analogous to the ADC(2) results, therefore, suggesting the geometries of these MECPs optimised at the ADC(2) level are relatively accurate. Moreover, the energy barriers between either the S_1_ or T_1_ minima and the corresponding MECPs are consistently described by both methods for the two photostabilising relaxation channels.

There is a slight variation between the methods for barriers in the photodestructive relaxation channels that involve the C(2)–O bond breaking in both the singlet and triplet manifolds. In the triplet pathway, the barrier increases to 0.4 eV relative to the 0.2 eV barrier computed at the ADC(2) level. NEVPT2 gives a barrier of 0.7 eV for the singlet pathway, which is 0.3 eV higher than the value calculated at the ADC(2) level. Although these barriers increase when computed at the NEVPT2 level, it is important to note that we do not present minimum energy paths and these results present upper bounds to the expected barrier heights. Furthermore, considering the excess energy acquired by the molecule during the absorption of a UV photon, the triplet C(2)–O bond breaking mechanism can still be considered as one of the major photorelaxation mechanisms of 2-TO.

In addition to these barrier differences between the two methods, the NEVPT_2_ method suggests that the $\text{π}\sigma_{\text{NH}}^{*}/{\text{S}}_{0}$ MECP in the N–H bond fission pathway can be found at a shorter N–H distance (1.91 Å) than as indicated by the ADC(2)/ MP2 methods (1.98 Å). Nevertheless, the shapes of the $\text{π}\sigma_{\text{NH}}^{*}$ PE are also qualitatively consistent at both levels of theory and we do not expect this to have any significant effect on the photodynamics. In fact the ADC(2)/MP2 methods are expected to be less reliable in the vicinity of S_1_/S_0_ MECPs.

## 4 Conclusions

In summary, we performed UV irradiation experiments and a comprehensive computational investigation of the photochemical properties of 2-thiooxazole (2-TO), which is considered as a prebiotically credible precursor of RNA nucleotides. Our experimental results demonstrated that 2-TO undergoes rapid photodegradation in a similar fashion to other oxazole molecules.^39,75^ However, the analysis of photoproducts was insufficient to determine whether the photodegradation of 2-TO operates through desulfurisation and subsequent photodecomposition of oxazole, or a different mechanism.

Our nonadiabatic dynamics simulations and explorations of excited-state PE surfaces using the ADC(2)/MP2 methods allowed us to clarify the origin of 2-TO photodegradation. In particular, we identified six primary photorelaxation channels of 2-TO. The dominant relaxation pathway was the photodestructive channel, which is associated with the πσ^*^/S_0_ MECP that proceeds with the C(2)–O bond breaking and occurs in both the singlet and triplet manifolds. This channel was followed by approximately 50% of the trajectories and is most likely the main cause of rapid 2-TO photodegradation. Thus, 2-TO undergoes direct and efficient photochemical rupture of the aromatic ring in a similar way to it’s oxazole derivatives which do not contain sulfur.^39,75^ This process is likely followed by subsequent radical recombination and hydrolysis reactions, which lead to the complex mixture of small molecule products observed with NMR. Our simulations also demonstrate the accessibility of a photostabilising channel in the singlet and triplet excited states of 2-TO. This channel proceeds through a n_S_π^*^/S_0_ MECP, where the C–S bond tilts out of the plane of the ring and there is slight ring puckering due to pyramidalisation of the N atom. However, even though we observed this photostabilising channel for 37% of the simulated trajectories, the prevalence of photochemical C(2)–O bond breaking eventually determines the very low photostability of 2-TO. It is worth emphasizing that all of the excited-state dynamics simulations and potential energy surface explorations were performed in the gas phase and, thus, did not perfectly reproduce the experimental conditions. Nevertheless, the unimolecular photochemical breaking of the C(2)–O bond is an ultrafast process driven by the excess vibrational energy gained by photoexcited 2-TO and should be readily available in aqueous environment. We anticipate that water molecules could play a primary role in the hydrolysis of the intermediate formed by photochemical rupture of the aromatic ring of 2-TO.

In order to validate the nonadiabatic dynamics simulations results, we recalculated the PE profiles for the primary photorelaxation mechanisms using the NEVPT2/SA-CASSCF multi-configurational approach. These results demonstrated excellent qualitative agreement with the ADC(2)/MP2 method used for excited-state dynamics simulations. The excited-state potential energy surfaces obtained at the NEVPT_2_ level were only systematically shifted with respect to the corresponding ADC(2)/MP2 results. This adds further confidence with respect to the accuracy of our nonadiabatic dynamics simulations and the identification of the photodegradation mechanism.

In conclusion, our computational and experimental results demonstrate that 2-TO is very photounstable and it would be prone to rapid photodegradation when exposed to UV light, which is necessary to promote the key selective photochemical transformations that could yield cytidine and uridine.^7,8,14–19^ Therefore, 2-TO could be a prebiotically credible precursor of RNA nucleotides only under conditions that could shield this compound from UV light. A possible scenario would require generation of 2-TO and subsequent reaction with glycer-aldehyde leading to oxazolidinone thione to occur in the dark. In fact, we have previously demonstrated that oxazolidinone thione is a highly photostable RNA precursor and could accumulate even during prolonged periods of UV irradiation.^52^ Alternatively, 2-TO could be partly shielded from UV light by the presence of much more photostable compounds that absorbs light in a similar spectral window, *e.g*. adenosine or 2,6-diaminopurine.^33,40^ A similar sun-screening effect was recently demonstrated for 2-aminooxazole in the presence of much more photostable 2-aminoimidazole.^40^ Such constraints on the synthesis and prebiotic scenario proposed by Stairs *et al*.^35^ could still enable the concomitant formation of pyrimidine and 8-oxopurine nucleotides under prebiotically plausible conditions. Nevertheless, further research is necessary to unequivocally establish whether 2-TO could act as a plausible intermediate of RNA nucleotides under continuous UV irradiation. Future spectroscopic studies of the unimolecular photochemistry of 2-TO, performed in low-temperature inert matrices, could also shed more light on the ring-opening mechanism as well as possible alternative photorelaxation mechanisms involving proton transfer and tautomerization as observed for related thiocarbonyl compounds, namely thiourea and thioacetamide.^78,79^

## Supplementary Material

Energy geometries

Supplementary information

## Figures and Tables

**Table 1:** Vertical excitations energies (in eV) of 2-TO obtained at the ADC(2)/aug-cc-pVTZ level of theory, assuming the ground-state minimum-energy geometry found using the MP2/aug-cc-pVTZ method. These are calculated in the gas phase and using COSMO

State	Transition	*E*_exc_ (eV)	*f*_osc_	*λ* (nm)
Gas phase				
S_1_	${\text{nπ}}_{\text{CS}}^{*}$	4.23	3.65 × 10^−5^	293.0
S_2_	${\text{nπ}}_{\text{CS}}^{*}$	4.64	0.364	267.4
S_3_	πσ^*^	5.06	0.022	245.1
S_4_	nσ*	5.56	0.024	223.0
S_5_	πσ^*^	5.81	3.57 × 10^−3^	213.3
S_6_	πσ^*^	6.03	0.031	205.5
COSMO				
S_1_	${\text{ππ}}_{\text{CS}}^{*}$	4.55	0.417	271.8
S_2_	${\text{nπ}}_{\text{CS}}^{*}$	4.61	1.88 × 10^−5^	261.0
S_3_	πσ^*^	5.43	0.066	226.1
S_4_	nσ*	6.10	0.060	200.8
S_5_	πσ^*^	6.17	4.53 × 10^−4^	197.5
S_6_	πσ^*^	6.33	0.013	192.1
