# Supplementary material for: Photochemistry of 2-thiooxazole: a plausible prebiotic precursor to RNA nucleotides
Source: Phys Chem Chem Phys. Author manuscript; Available in PMC 2022 Oct 11. (PMC7613695; doi:10.1039/d2cp03167a)
Supplement: Supplementary information [file EMS155319-supplement-Supplementary_information.pdf]

Electronic Supplementary Material (ESI) for Physical Chemistry Chemical Physics.
This journal is © the Owner Societies 2022

Electronic Supplementary Information (ESI) for Photochemistry of 2-thiooxazole: a plausible prebiotic precursor to RNA nucleosides

Lauren Bertram,[a] Samuel J. Roberts,[b,c] Matthew W. Powner,[b] and Rafał Szabla*[a,d]

11th July 2022

Theoretical section

Excited-state properties

Table S1 Magnitude (μ) and x,y,z (μ_x, μ_y, μ_z) components of the unrelaxed dipole moment vectors computed at the ADC(2)/aug-cc-pVTZ level of theory, assuming the ground-state minimum energy geometry was found using the MP2/aug-cc-pVTZ method.

State	Transition	μ / D	μ_x	μ_y	μ_z
S_1	$n\pi^*_{CS}$	2.11	0.148	0.209	0.791
S_2	$\pi\pi^*_{CS}$	5.85	-1.20	-0.771	1.80
S_3	$\pi\sigma^*$	5.91	-1.01	-1.02	-1.83
S_4	$n\sigma^*$	5.93	-0.289	-0.489	-2.26
S_5	$n\sigma^*$	9.28	2.75	2.09	-1.19
S_6	$n\sigma^*$	2.79	0.813	0.687	0.273

Table S2 Magnitude (μ) and x,y,z (μ_x, μ_y, μ_z) components of the relaxed dipole moment vectors computed at the ADC(2)/aug-cc-pVTZ level of theory, assuming the ground-state minimum energy geometry was found using the MP2/aug-cc-pVTZ method.

State	Transition	μ / D	μ_x	μ_y	μ_z
GS	-	5.71	-1.11	-0.695	1.82
S_1	$n\pi^*_{CS}$	2.85	-0.253	-0.0825	1.09
S_2	$\pi\pi^*_{CS}$	6.32	-1.35	-0.882	1.89
S_3	$\pi\sigma^*$	5.32	-1.05	-1.02	-1.51
S_4	$n\sigma^*$	4.97	-0.872	-0.877	-1.52
S_5	$n\sigma^*$	8.60	2.56	1.96	-1.03
S_6	$n\sigma^*$	2.46	0.678	0.588	0.360

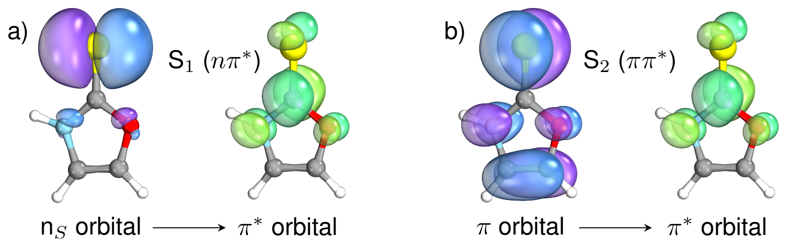

Figure S1 Molecular orbitals in the Franck-Condon region showing the character of the electronic transitions for the two lowest-lying singlet excited states.

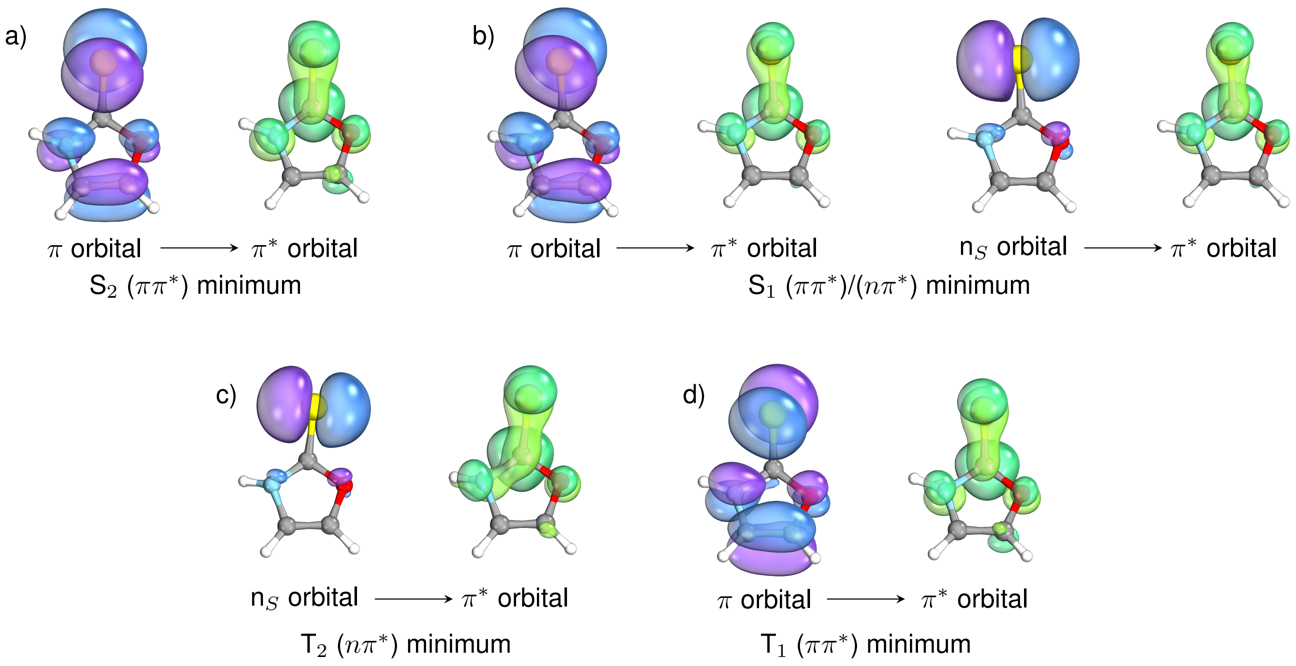

Figure S2 Molecular orbitals corresponding to the electronic transitions of the S_2, S_1, T_2 and T_1 minima.

Potential energy (PE) profiles of the minor photorelaxation channels

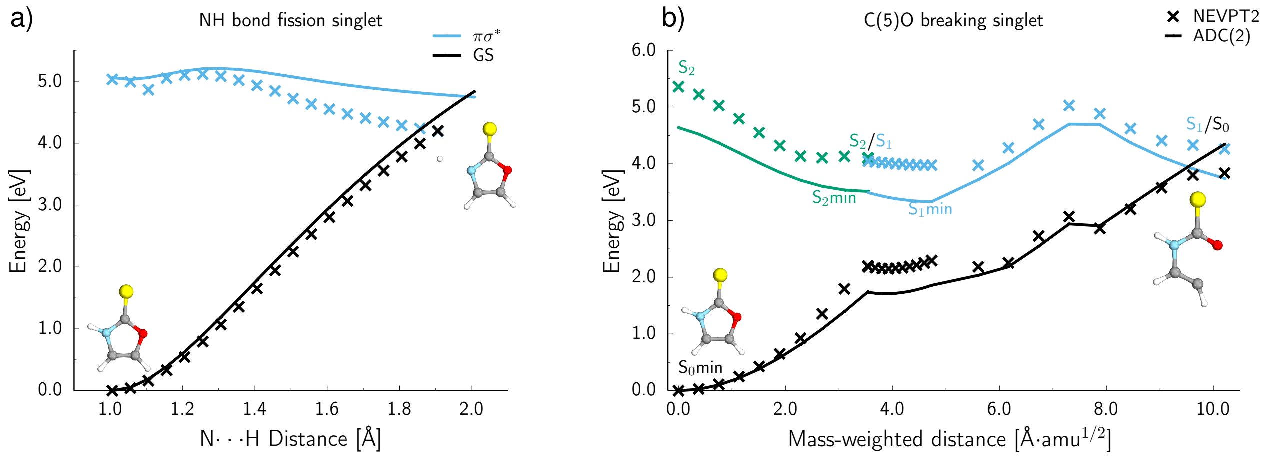

Figure S3 The potential energy (PE) profiles of the two minor deactivation channels of 2-TO. The lines present PE surfaces performed at MP2/ADC(2) level for (a) and at the SCS-MP2/SCS-ADC(2) level for (b), all with the aug-cc-pVTZ basis set. The crosses represent the PE surfaces performed at the NEVPT2/SA-CASSCF(10,8) with the aug-cc-pVDZ basis set for (a) and the cc-pVTZ basis set for (b).

Experimental section

General experimental

All compounds were obtained from: Sigma Aldrich, Alfa Aesar and Fluorochem unless specified. UV irradiations were performed using a Rayonet RPR-200 with 6 × RPR-2547A lamps or with 6 × RPR-3000A lamps. Solution pH values were measured with a Corning pH meter 430 with a Fischerbrand FB68801 semi-micro pH probe or a Mettler Toledo Seven Compact pH meter with a Mettler Toledo InLab semi-micro pH probe. Water (H_2O) refers to deionised water produced by an Elga Option 3 purification system unless specified otherwise. Melting points were determined using an Electrothermal standard digital apparatus. Melting points are quoted to the nearest °C and are uncorrected. Bruker NMR spectrometer AVANCE III 600 equipped with a Bruker 5 mm cryoprobe (600 MHz) and Bruker NMR spectrometer AVANCE Neo 700 equipped with a room-temperature 5mm BBO probe were used to provide ^{1}H NMR data. Water supressed ^{1}H NMR spectra were obtained using a suppression pulse sequence (noesygppr1d, Bruker) and all spectra were recorded at 298 K. Spectra reported in H_2O were performed with no lock. All reported chemical shifts (δ) are given in parts per million (ppm)

relative to residual solvent peaks, and ^{1}H spectra calibrated using the residual solvent peaks relative shift to TMS. Coupling constants (J) are given in Hertz (Hz). The following abbreviations refer to spin multiplicities: s (singlet); d (doublet); t (triplet); m (multiplet); or any combination of these. Diastereotopic geminal (AB) spin systems coupled to an additional nucleus are reported as ABX. NMR data are stated as follows: chemical shift (number of protons, multiplicity, coupling constants (J), nuclear assignment). Ultraviolet spectra (UV) were recorded on a Shimadzu UV-1800 UV spectrophotometer. Absorption maxima are reported in wavelength (nm). Infrared spectra (IR) were recorded on a Shimadzu IR Tracer 100 FT-IR spectrometer. Absorption maxima are reported in wavenumber (cm^{-1}).

Methods

General irradiation procedure

A solution of specified heterocycle (6.00 mmol, 2 mM in H_2O) was adjusted to the desired pH and then degassed with a stream of nitrogen for 2 h in a quartz tube. The tube was sealed with a rubber septum, flushed with argon, and then kept under positive pressure argon atmosphere. The solution was irradiated using the specified UV lamps for 16 h at 38 °C, then left to relax for 1 h. The solution was analysed by NMR spectroscopy. A solution of pentaerythritol (0.100 M, 50.0 μL, 5.00 μmol in H_2O/D_2O 9:1) was added as an internal NMR standard and NMR spectra were then reacquired.

Irradiation of 2-thiooxazole

2-Thio-oxazole was submitted to the general irradiation procedure with 254 nm lamps at pH 3, 6.5 or 12, or 300 nm lamps at pH 6.5. Analyses of NMR spectra showed consumption of starting material in all cases (except 300nm, pH 6.5, 29% 2-thio-oxazole) and no formation of oxazole (Supplementary Fig. S4).

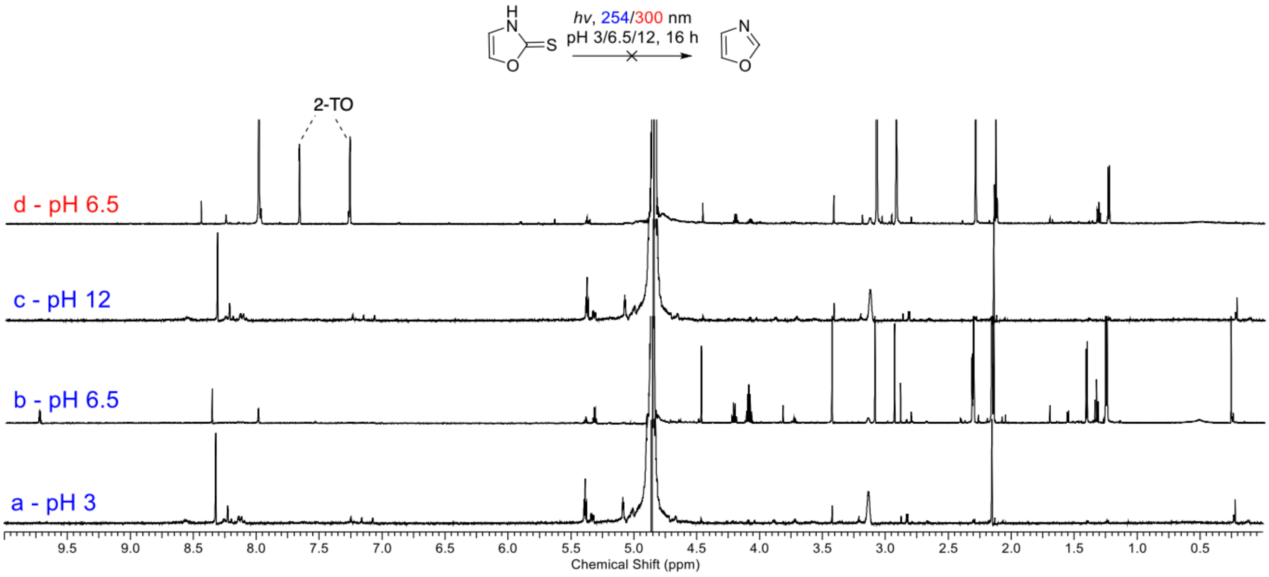

Figure S4 ^{1}H NMR (600 MHz, H_2O, noesygppr1d, 0.0–10.0 ppm) spectra to show the irradiation of 2-thio-oxazole (2-TO, 2 mM). a. Spectra to show the products of 254 nm irradiation after 16 h at pH 3. b. Spectra to show the products of 254 nm irradiation after 16 h at pH 6.5. c. Spectra to show the products of 254 nm irradiation after 16 h at pH 12. d. Spectra to show the products of 300 nm irradiation after 16 h at pH 6.5.

Control irradiation of oxazole

Oxazole was submitted to the general irradiation procedure with 254 nm lamps at 6.5. Analyses of NMR spectra showed complete consumption of starting material in all cases (Supplementary Fig. S5).

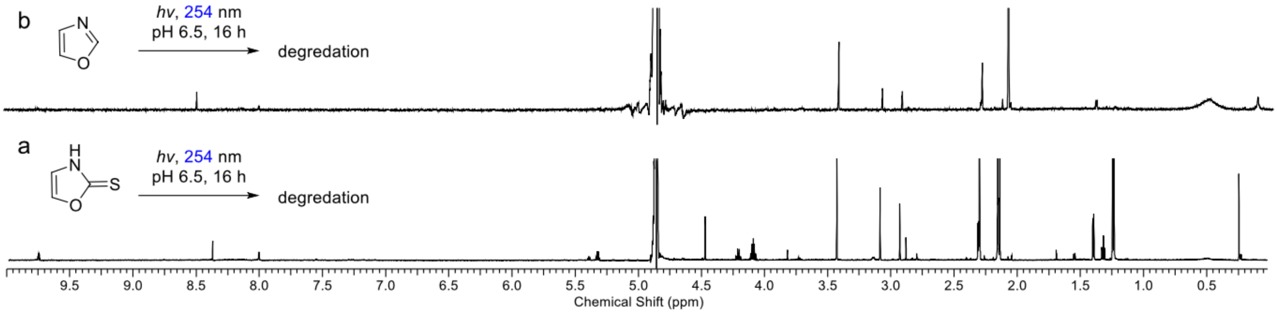

Figure S5 ^{1}H NMR (600 MHz, H_2O, noesygppr1d, 0.0–10.0 ppm) spectra to compare the irradiation (254 nm, 16 h) of oxazole (2 mM) and 2-thio-oxazole (2 mM). a. Spectra to show the products of 254 nm irradiation of 2-thio-oxazole after 16 h at pH 6.5. b. Spectra to show the products of 254 nm irradiation of oxazole after 16 h at pH 6.5.

Synthesis and characterisation of 2-thiooxazole (2-TO)

2-Thio-oxazole was synthesised following a literature protocol and the compounds data was found to match the literature values.[1]

δ_H (600 MHz, D_2O): 7.33 (1H, d, J = 4.5 Hz, H4), 7.05 (1H, d, J = 4.5 Hz, H5); δ_C (150 MHz, D_2O): 188.1 (C2), 130.0 (C4), 117.2 (C5). HRMS (ESI): C3H3NS2 predicted mass 100.9930 [M+H], found 100.9930. HRMS (ESI): $C_3H_3NS_2$ predicted mass 100.9930 [M+H], found 100.9930. IR (cm^{-1}): 3117 (NH), 1587 (C=C), 1478 (C=S). M.P.: seen 140-144 °C. λ_{max} (nm) = 263.

2-Thiooxazole spectra

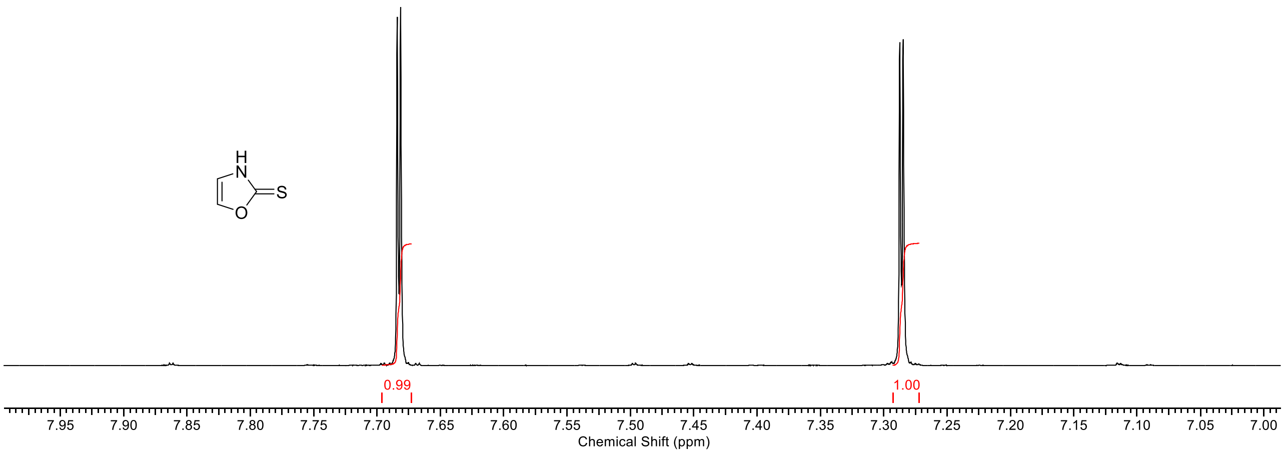

Figure S6 ^{1}H NMR (600 MHz, D_2O, 7.0–8.0 ppm) spectra of 2-thiooxazole.

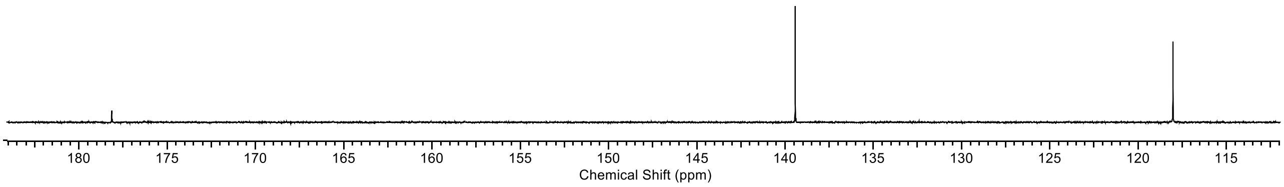

Figure S7 ^{13}C NMR (150 MHz, D_2O, 112.0–184.0 ppm) spectra of 2-thiooxazole.

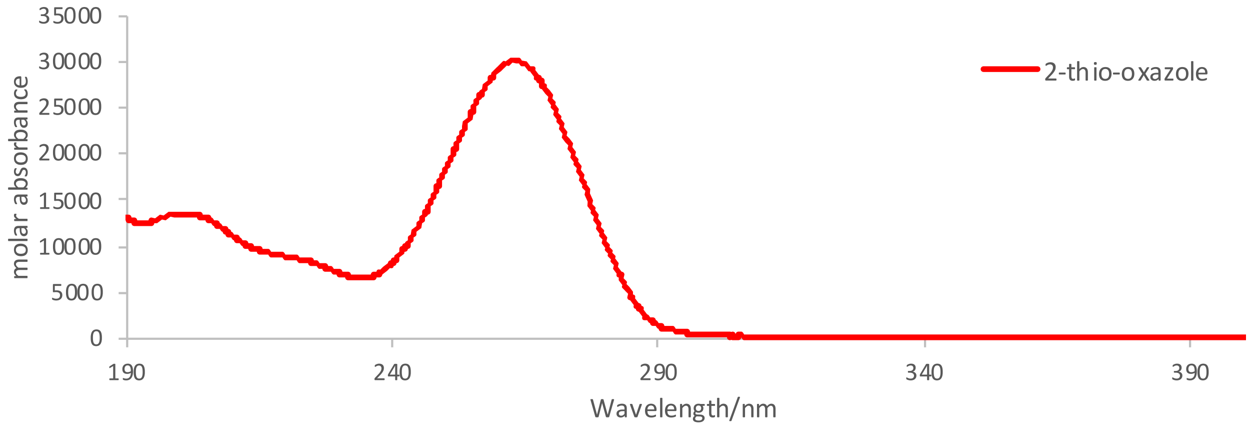

Figure S8 UV/Vis spectra (400-190 nm, H_2O) of 2-thiooxazole.

References

[1] S. Stairs, A. Nikmal, D.-K. Bučar, S.-L. Zheng, J. W. Szostak and M. W. Powner, *Nat Commun*, 2017, **8**, 15270.
